# Age at Menarche, Menstrual Characteristics, and Risk of Preeclampsia

**DOI:** 10.5402/2011/472083

**Published:** 2011-12-29

**Authors:** Dejene F. Abetew, Daniel A. Enquobahrie, Michal Dishi, Carole B. Rudra, Raymond S. Miller, Michelle A. Williams

**Affiliations:** ^1^Center for Perinatal Studies, Swedish Medical Center, Seattle, WA 98104, USA; ^2^Department of Epidemiology, University of Washington, Seattle, WA 98195, USA; ^3^Department of Social and Preventive Medicine, The State University of New York at Buffalo, Buffalo, NY 14214-8001, USA

## Abstract

We examined associations of age at menarche and menstrual characteristics with the risk of preeclampsia among participants (*n* = 3,365) of a pregnancy cohort study. Data were collected using in-person interviews and medical record abstraction. Logistic regression was used to estimate adjusted odds ratio (OR) and 95% confidence interval (95% CI). There was a significant inverse association between age at menarche and risk of preeclampsia (*P* value for trend < 0.05). Association of long cycle length (>36 days) with higher risk of preeclampsia was present only among women who had prepregnancy body mass index <25 kg/m^2^ (interaction *P* value = 0.04). Early menarche is associated with higher risk of preeclampsia. Prepregnancy weight may modify associations of long menstrual cycles with risk of preeclampsia.

## 1. Introduction

Menarche, an important milestone of sexual development, signals the end of puberty and the beginning of reproductive life in females [1, 2]. Both early and delayed menarche have been associated with increased cardiovascular disease (CVD) risk factors (including metabolic syndrome) and disease in adolescent girls and young women [1, 3–7]. Further more, women with long and irregular menstrual cycles have been shown to have higher risk for CVD and type 2 diabetes [5, 7, 8].

Preeclampsia, a multisystem pregnancy disorder, is characterized by hypertension and proteinuria that develop after 20 weeks of gestation [9–14]. The precise pathophysiologic mechanisms of preeclampsia remain unknown and are subjects of extensive research [9–12, 15–20]. Since previously identified risk factors of preeclampsia (including maternal obesity, insulin resistance, and other hormonal factors) [15, 18] are potentially associated with early menarche, delayed menarche and/or menstrual irregularities [4, 5], age at menarche, and menstrual characteristics may be associated with risk of preeclampsia. Previous reports on associations of menstrual characteristics with preeclampsia were based on case-control studies [5]. Among a well-characterized pregnancy cohort, we investigated relationships between age at menarche and menstrual characteristics with risk of preeclampsia. We also examined whether prepregnancy body mass index (BMI), adult weight gain, or maternal birth weight, a marker of intrauterine development that has been related to reproductive outcomes [21–24], modified these associations.

## 2. Methods

### 2.1. Study Population

Study participants were drawn from participants of the Omega study [9, 25]. Briefly, the study population comprised of women attending prenatal care clinics affiliated with Swedish Medical Center (SMC) in Seattle, WA, and Tacoma General Hospital (TGH) in Tacoma, WA. Women were eligible if they initiated prenatal care before 20 weeks of pregnancy, were >18 years old, were able to speak and read English, plan to carry the pregnancy to term, or planned to deliver at either of the two research hospitals. During 1996–2008, 5,063 eligible women were approached and 4,000 (79%) participants were enrolled in the study. Of these, participants who moved or delivered elsewhere (*n* = 151), delivered before 20 weeks (*n* = 43), had prior chronic hypertension (*n* = 167), and had no available information on menstrual age (*n* = 274) or menstrual characteristics were excluded from current analyses (*n* = 3, 365). Study protocols were approved by the Institutional Review Boards of SMC and TGH. All participants provided written informed consent.

### 2.2. Data Collection

Using standardized questionnaires administered by a trained interviewer at or near the time of enrollment, we gathered information on age at menarche, menstrual characteristics, and other covariates including maternal sociodemographic factors, medical/reproductive histories, prepregnancy BMI, adult weight gain (differences in weight at age 18 and prepregnancy weight), and maternal birth weight. After delivery, maternal and infant medical records were reviewed for information on the course and outcomes of pregnancy.

Preeclampsia was defined using American College of Obstetricians and Gynecologists (ACOG) guidelines [26] as sustained pregnancy-induced hypertension with proteinuria. Hypertension was defined as sustained elevated blood pressure (BP) readings of 140/90 mm Hg (with readings taking place 6 hours apart) after 20 weeks of gestation. Proteinuria was defined as protein concentration of ≥30 mg/dL on ≥2 random urine specimen collected 4 hours apart.

We gathered information on menstrual cycle characteristics including (a) age at menarche (the interviewer asked “At what age did you have your first period?”), (b) irregular menses after menarche (the interviewer asked “During the first year after starting your menstrual periods, did your periods become regular? That is, could you predict within one week when your next menstrual period would begin?” and “Have your period ever been regular without using birth control pills, injection, or implants?”), and (c) usual cycle length (the interviewer asked “On average, how often did you have your menstrual period? That is, how many days were there between the first day of one menstrual period and the first day of the next?”).

### 2.3. Statistical Analysis

We examined general characteristics of the study population using mean (standard deviation) for continuous variables and numbers (%) for categorical variables. We used unadjusted and multivariable adjusted logistic regression analyses to compute odds ratio (OR) and 95% confidence interval (95% CI). We used *a priori* identified indicator variables to categorize age at menarche (≤11, 12 13, 14, and ≥15 years) and cycle length (≤24, 25–30, 31–35, and ≥36 days) [5]. Categories of age 13 years and 25–30 days were used as references for age at menarche and cycle length categories, respectively. Potential confounders that resulted in >10% difference in estimated ORs (comparing unadjusted and adjusted regression coefficients) were considered as confounders and were retained in multivariable models. The first adjusted model included variables for maternal age, race/ethnicity, parity, maternal birth weight, and pregestational diabetes. The second adjusted model included variables for prepregnancy BMI in addition to variables included in Model 1. We also evaluated potential effect modification of the relationship between early age at menarche and menstrual cycle length with preeclampsia risk by prepregnancy overweight/obesity status (BMI ≥ 25 kg/m^2^), adult weight gain, and maternal birth weight. For these analyses, we defined early menarche as age at menarche ≤11 years and long menstrual length as menstrual cycle ≥36 days. We used both interaction terms and stratified analyses to evaluate the presence of effect modification [27]. All analyses were conducted using SPSS version 14. Statistical significance was defined as two-sided *P* < 0.05.

## 3. Results

Participants, 32.6 years old on average, were predominantly white (86.4%) (Table 1). Among 3,365 participants of the cohort, 80 (2.4%) developed preeclampsia. We observed a statistically nonsignificant higher risk of preeclampsia (unadjusted OR: 1.56, 95% CI: 0.76–3.19) among participants with long cycle length (≥36 days) compared with participants with normal cycle length (25–30 days) (Table 2). However, this relationship was not present after adjustment for confounders. We did not find associations of irregular menses with risk of preeclampsia. There was a significant inverse association between age at menarche and risk of preeclampsia (*P* value for trend < 0.05) (Table 2). While the increased risk of preeclampsia associated with age at menarche ≤11 years, compared with the referent (age 13), did not reach statistical significance, women who had menarche at ≤10 years of age (*n* = 8 preeclampsia cases and *n* = 106 controls) had a 3-fold increase in risk of preeclampsia compared with women who had menarche at age 13 years (adjusted OR: 2.99, 95% CI 1.24–7.21) (not shown).

In effect modification analyses, we observed significant interaction between prepregnancy BMI and cycle length (*P* value for interaction = 0.04) (Table 3). The association between longer cycle length and higher risk of preeclampsia was present only among women who had prepregnancy BMI < 25 kg/m^2^ (OR: 2.56, 95% CI: 1.12–5.88) but not among women who had prepregnancy BMI ≥ 25 kg/m^2^ (OR: 0.44, 95% CI: 0.10–1.84). The association between early age at menarche (≤11 years) and risk of preeclampsia was significantly higher and statistically significant among women who had birth weight ≥2.5 kg (OR: 3.57, 95% CI: 1.07–11.93) compared with the association among women who had birth weight <2.5 kg (OR: 1.20, 95% CI: 0.45–3.18), although the interaction term was not statistically significant (*P* value > 0.05). Women who were overweight and had early menarche (≤11 years) had a 4-fold increase in risk of preeclampsia compared with women who were not overweight and had menarche after age 11 (95% CI: 2.24–8.66). Women who gained ≥5.0 kg in adulthood and did not have long cycles or early menarche had 4- to 5-fold higher risks of preeclampsia that were statistically significant compared with women who gained <5 kg in adulthood and did not have long cycles (OR: 4.33, 95% CI: 1.63–11.45) or early age at menarche (OR: 5.28, 95% CI: 2.42–11.56), respectively (*P* values of interaction > 0.05).

## 4. Discussion

We observed associations of early age at menarche with increased risk of preeclampsia and evidence of effect modification of the cycle length and risk of preeclampsia association by maternal prepregnancy body mass index. We also noted potential effect modifications of the early menarche and risk of preeclampsia association by maternal birth weight. Similarly, adult weight gain appeared to modify the associations of age at menarche with risk of preeclampsia and long menstrual cycles with risk of preeclampsia, though these interactions were not statistically significant.

Previously, early menarche (≤12 years) has been associated with increased risk of CVD events [1]; investigators have hypothesized that observed associations are potentially mediated by increased adiposity associated with early menarche [1]. In the current study, we found an inverse relationship between age at menarche and increased risk of preeclampsia. In a case-control study, also conducted in Seattle, WA, Rudra and colleagues reported nonsignificant 3-fold increase in risk of preeclampsia among overweight women with longer menstrual cycle length (OR: 3.11, 95% CI: 0.62–15)[5], similar to our findings. The evidence from the current study is stronger since it is based on a study population from a prospective cohort study where maternal recall of age at menarche and menstrual characteristics is not influenced by the outcomes of the pregnancy. Previously, adult weight gain of ≥10 kg has also been associated with a 5-fold increased risk of preeclampsia (OR: 5.1, 95% CI: 2.2–12.2) [25]. However, its role in the relationships of age at menarche and cycle length with risk of preeclampsia has not been well investigated. In the current study, we provide some evidence of potential synergistic interactions between these risk factors.

Girls who mature early (have early age at menarche) tend to be heavier (overweight or obese) as adults [1, 6, 28]. It has been suggested that early maturing girls may have a longer period of positive energy balance or other endocrine factors that influence both the rate of sexual maturation and the accumulation of body fat [6]. Obesity is associated with cardiometabolic abnormalities, including inflammation, and oxidative stress insulin resistance, which have been related to preeclampsia and other pregnancy complications. A number of biomarkers (e.g., C-reactive protein, tumor necrosis alpha) or pathomechanisms (e.g., endothelial dysfunction and dyslipidemia) that have been shown to be associated with both overweight/obesity status and preeclampsia support this evidence [4, 9, 16, 19, 28–30].

Birth weight is an indicator of intrauterine growth, a period of critical growth and programming [21–24]. Thus, maternal birth weight may be directly related to pathophysiologic changes that occur during pregnancy, including those that relate to risk of preeclampsia [21, 22]. In addition, maternal birth weight may play an indirect role in preeclampsia risk through its influence on other risk factors of preeclampsia (e.g., obesity).

Some limitations of our study deserve mention. While we collected and had access to information about maternal characteristics and other covariates, we are aware of the fact that self-reported medical histories may be subject to recall bias and misclassification. However, our prospective study design would assure that the potential misclassification is not conditional on the diagnosis of preeclampsia. Although we adjusted for several potential confounders, we cannot exclude the possibility of residual confounding. Finally, we cannot comment on the sequential influence of childhood and pubertal obesity on menstrual characteristics and eventual risk of preeclampsia.

## 5. Conclusion

We have shown that early onset of menarche is associated with an increased risk of preeclampsia and prepregnancy weight modifies associations of cycle length with risk of preeclampsia. Prepregnancy weight, adult weight gain, and maternal birth weight all appear to influence preeclampsia risk associated with early age at menarche and longer menstrual cycles. Future larger studies that investigate this research area may create opportunities for prevention and early intervention of preeclampsia.

## Figures and Tables

**Table 1:** Age at menarche and selected characteristics of the study population.

Characteristics	Entire cohort	Age of menarche (years)				
	(*n* = 3365)	≤11 (*n* = 487)	12 (*n* = 881)	13 (*n* = 1048)	14 (*n* = 493)	≥15 (*n* = 456)
Maternal age, years^a^	32.6 ± 4.5	31.9 ± 4.9	32.6 ± 4.5	32.6 ± 4.2	32.7 ± 4.3	33.1 ± 4.2
<20	20 (0.6)	9 (1.8)	1 (0.1)	6 (0.6)	2 (0.4)	2 (0.4)
20–34	2229 (66.2)	333 (68.4)	587 (66.6)	706 (67.4)	329 (66.7)	274 (60.1)
35+	1116 (33.2)	145 (29.8)	293 (33.3)	336 (32.1)	162 (32.9)	180 (39.5)
Maternal White race	2909 (86.4)	380 (78)	752 (85.4)	922 (88)	447 (90.7)	408 (89.5)
Post-high-school education	3237 (96.2)	457 (93.8)	843 (95.7)	1016 (96.9)	478 (97)	443 (97.1)
Nulliparous	2138 (63.5)	309 (63.4)	559 (63.5)	686 (65.5)	301 (61.1)	283 (62.1)
Smoked during pregnancy	200 (5.9)	38 (7.8)	52 (5.9)	53 (5.1)	28 (5.7)	29 (6.4)
Prepregnancy BMI, kg/m² ^a^	23.4 ± 4.7	25.3 ± 5.9	23.9 ± 4.9	22.9 ± 4.1	22.4 ± 3.8	22.3 ± 3.9
<18.5	147 (4.4)	6 (1.2)	30 (3.4)	45 (4.3)	32 (6.5)	34 (7.5)
18.5–24.9	2434 (72.3)	293 (60.2)	607 (68.9)	799 (76.2)	379 (76.9)	356 (78.1)
25–29.9	526 (15.6)	109 (22.4)	167 (19)	137 (13.1)	63 (12.8)	50 (11)
30+	258 (7.7)	79 (16.2)	77 (8.7)	67 (6.4)	19 (3.9)	16 (3.5)
Maternal birth weight (kg)^a^	3.3 ± (0.5)	3.2 ± (0.5)	3.3 ± (0.5)	3.3 ± (0.5)	3.3 ± (0.5)	3.3 ± 0.6
<2.50	271 (8.1)	44 (9)	72 (8.2)	70 (6.7)	41 (8.3)	44 (9.6)
2.50–3.9	2735 (81.3)	403 (82.8)	704 (79.9)	861 (82.2)	405 (82.2)	362 (79.4)
>4.0	359 (10.7)	40 (8.2)	105 (11.9)	117 (11.2)	47 (9.5)	50 (11)
Polycystic ovary syndrome	95 (2.8)	12 (2.5)	32 (3.6)	17 (1.6)	18 (3.7)	16 (3.5)
FH of hypertension	1652 (49.1)	259 (53.2)	459 (52.1)	473 (45.1)	240 (48.7)	221(48.5)
FH of diabetes	488 (14.5)	86 (17.7)	126 (14.3)	149 (14.2)	63 (12.8)	64 (14)
Adult weight change, kg^a,b^	7.5 ± 1.0	9.9 ± 12.6	8.1 ± 10.8	6.8 ± 9.3	6.2 ± 8.5	6.5 ± 7.7
<−2.5	263 (7.9)	34 (7.1)	75 (8.6)	81 (7.8)	38 (7.8)	35 (7.8)
−2.5–2.5	773 (23.3)	92 (19.3)	197 (22.7)	260 (25.2)	126 (25.9)	98 (21.9)
2.5–4.9	553 (16.7)	73 (15.3)	138 (15.9)	165 (16.0)	101(20.7)	76 (17.0)
5.00–9.9	776 (23.4)	91 (19.1)	180 (20.7)	263 (25.5)	106 (21.8)	136 (30.4)
>10.0	948 (28.6)	187 (39.2)	278 (32.0)	264 (25.6)	116 (23.8)	103 (23.0)
Leisure time exercise during pregnancy	2933 (87.2)	434 (89.1)	758 (86.0)	920 (87.8)	428 (86.8)	393 (86.2)
History of infertility problem	470 (14.0)	70 (14.4)	124 (14.1)	133 (12.7)	64 (13.0)	79 (17.3)
BMI 18 years^a^	20.6 ± 2.9	21.7 ± 3.5	20.9 ± 2.8	20.5 ± 2.7	20.1 ± 2.6	19.9 ± 2.8
<18.5	615 (18.6)	53 (11.1)	136 (15.7)	174 (16.8)	120 (24.6)	132 (29.5)
18.5–24.9	2493 (75.2)	367 (76.8)	678 (78.2)	804 (77.7)	347 (71.3)	297 (66.3)
25.0–29.9	157 (4.7)	39 (8.2)	41 (4.7)	45 (4.3)	16 (3.3)	16 (3.6)
>30.0	50 (1.5)	19 (4.0)	12 (1.4)	12 (1.2)	4 (0.8)	3 (0.7)

^a^Mean (SD) otherwise *n* (%).

^b^Weight change from age 18 to pregnancy.

BMI: body mass index (kg/m²), FH: family history.

**Table 2:** Association (odds ratio (OR), 95% (CI)) between menstrual cycle characteristics and risk of preeclampsia.

Characteristics	Cases	Noncases	Unadjusted		Adjusted Model 1		Adjusted Model 2	
			OR	95% (CI)	OR	95% (CI)	OR	95% (CI)
Usual menstrual cycle length (days)								
25–30	55 (68.8)	2383 (73.8)	1.00	Referent	1.00	Referent	1.00	Referent
<24	4 (5.0)	148 (4.6)	1.17	0.42–3.27	0.62	0.30–1.29	0.72	0.34–1.49
31–35	12 (15.0)	449 (13.9)	1.16	0.61–2.18	0.78	0.23–2.59	0.88	0.26–3.00
≥36	9 (11.3)	250 (7.7)	1.56	0.76–3.19	0.74	0.31–1.80	0.87	0.36–2.14
Irregular menses								
No	67 (83.8)	2766 (85.2)	1.00	Referent	1.00	Referent	1.00	Referent
Yes	13 (16.3)	482 (14.8)	0.89	(0.49–1.63)	0.91	0.49–1.68	1.01	0.54–1.90
Age at menarche (years)								
≤11	17 (21.3)	464 (14.3)	1.69	0.88–3.21	1.77	0.92–3.38	1.30	0.67–2.53*
12	23 (28.8)	847 (26.1)	1.25	0.69–2.26	1.29	0.71–2.34	1.15	0.63–2.10*
13	22 (27.5)	1015 (31.3)	1.00	Referent	1.00	Referent	1.00	Referent*
14	10 (12.5)	480 (14.8)	0.96	0.45–2.04	0.97	0.45–2.08	1.01	0.47–2.18*
≥15	8 (10.0)	442 (13.6)	0.83	0.36–1.89	0.83	0.36–1.90	0.93	0.41–2.12*

Model 1 is adjusted for maternal age, race/ethnicity, parity, maternal birth weight, and pregestational diabetes.

Model 2 includes additional adjustment for maternal prepregnancy body mass index.

*Trend *P* value < 0.05.

**Table 3:** Menstrual characteristics, age at menarche, body weight measurements, and the risk of preeclampsia.

Stratifying covariates and exposure of interest	Cases	Noncases	Stratified by BMI		Joint models	
			Adj. OR	95% (CI)	Adj. OR	95% (CI)
Prepregnancy BMI and long cycle length^1^						
<25 kg/m^2^, <36 days	35 (43.8)	2327 (71.6)	1.00	Referent	1.00	Referent
<25 kg/m^2^, ≥36 days	7 (8.8)	187 (5.8)	**2.56**	**1.12–5.88**	**2.50**	**1.09–5.72**
≥25 kg/m^2^, <36 days	36 (45.0)	653 (20.1)	1.00	Referent	**3.78**	**2.35–6.08**
≥25 kg/m^2^, ≥36 days	2 (2.5)	81 (2.5)	0.44	0.10–1.84	1.67	0.40–7.09
Prepregnancy BMI and early age at menarche						
<25 kg/m^2^, >11 years	37 (46.3)	2223 (68.4)	1.00	Referent	Referent	Referent
<25 kg/m^2^, ≤11 years	5 (6.3)	291 (9.0)	1.13	0.44–2.92	1.08	0.42–2.76
≥25 kg/m^2^, >11 years	26 (32.5)	561 (17.3)	1.00	Referent	**2.86**	**1.71–4.77**
≥25 kg/m^2^, ≤11 years	12 (15.0)	173 (5.3)	1.48	0.72–3.02	**4.41**	**2.24–8.66**
Maternal birth weight and long cycle length						
≥2500 gr, <36 days	64 (80.0)	2755 (84.8)	1.00	Referent	Referent	Referent
≥2500 gr, ≥36 days	7 (8.8)	225 (6.9)	1.36	0.61–3.00	1.36	0.31–3.00
<2500 gr, <36 days	2 (2.5)	33 (1.0)	1.00	Referent	2.65	0.62–11.31
<2500 gr, ≥36 days	7 (8.8)	235 (7.2)	0.53	0.10–2.84	1.28	0.58–2.83
Maternal birth weight and early age at menarche						
≥2500 gr, >11 years	57 (71.3)	2566 (79.0)	1.00	Referent	Referent	Referent
≥2500 gr, ≤11 years	3 (3.8)	40 (1.2)	**3.57**	**1.06–11.96**	**3.57**	**1.07–11.93**
<2500 gr, >11 years	6 (7.5)	218 (6.7)	1.00	Referent	1.26	0.54–2.95
<2500 gr, ≤11 years	14 (17.5)	424 (13.1)	1.20	0.45–3.18	1.54	0.85–2.80
Adult weight gain^2^ and long usual cycle length						
<5.0 kg, <36 days	14 (17.5)	1441 (44.4)	1.00	Referent	Referent	Referent
<5.0 kg, ≥36 days	3 (3.8)	122 (3.8)	2.71	0.76–9.72	2.47	0.70–8.73
≥5.0 kg, <36 days	57 (71.3)	1539 (47.4)	1.00	Referent	**3.87**	**2.14–6.98**
≥5.0 kg, ≥36 days	6 (7.5)	146 (4.5)	1.12	0.47–2.64	**4.33**	**1.63–11.45**
Adult weight gain and early age at menarche						
<5.0 kg, >11 years	13 (16.3)	1369 (42.2)	1.00	Referent	Referent	Referent
<5.0 kg, ≤11 years	4 (5.0)	194 (6.0)	2.40	0.77–7.52	2.26	0.73–7.03
≥5.0 kg, >11 years	50 (62.5)	1415 (43.6)	1.00	Referent	**3.79**	**2.05–7.03**
≥5.0 kg, ≤11 years	13 (16.3)	270 (8.3)	1.36	0.47–2.64	**5.28**	**2.41–11.56**

All models are adjusted for age, race, family history of diabetes, and physical activity.

^1^Interaction *P* value 0.04.

^2^Adult weight gain: difference between weight at age 18 and prepregnancy weight.
